# Cholera as Influenced by Locality

**Published:** 1849-09

**Authors:** 


### CHOLERA AS INFLUENCED BY LOCALITY.

In our last number, we fell into an error, which we deem it proper to correct. We stated that the point, in Nashville, where cholera was most fatal, was the hill south of the University. We have been informed that it was not on the *hill*, but in a valley, in a southwest direction from the University, that the disease prevailed with such malignity.—

While ravaging this neighborhood, it scarcely prevailed in the old quarters of the city.

On this subject, we find some interesting facts in a St. Louis paper. The writer remarks: "Some particular spots have been particularly known as infected districts, and have been nearly swept of their population. At the corner of Washington avenue and Ninth street, opposite to the late ground of the caravan, several houses were left tenantless, the disease having taken off every one of the inmates. In a part of the city known as Shephard's grave yard, there have been from 80 to 100 deaths by the disease. The latter place is described as abounding in aliment for pestilence. In the former, also, there is a great accumulation of offensive matter."

Papers of a later date, from that city, however, furnish facts of an opposite character. They state that in the localities where cholera was for a time so fatal, the pestilence has ceased, and was prevailing in districts inhabited by people in the best circumstances. While thus desolating the city, and attacking and carrying off citizens of note, it was sparing the immigrants in their crowded, filthy, wretched quarters at quarantine, on the river a short distance below.

On another point, the St. Louis Union, from which we have quoted, gives some affecting statistics. In the whole history of cholera we are not sure that we have remarked anything more affecting than the facts stated in the following paragraphs:

It has already been stated in our paper that the number of deaths in this city for the week ending July 2d, were 903; of these the deaths by cholera were 619; those of five years and under, 230. In the previous week, ending June 25th, the total was 764; by cholera, 595; of five years and under, 171.

The most remarkable feature of this malignant pestilence is, that the greatest number of its victims are from those in middle life—the period most exempt of all others from death by ordinary diseases—those in the vigor and strength of manhood—the father and mother, at the period when the young family is most numerous and most needing aid and guidance, are the principal food for the destroyer. In the first week, ending 25th ult., those between 20 and 40 years were 251, or exceeding five-twelfths of the whole number, and exceeding the deaths of the same ages by other diseases nearly eight times. While of 5 years and under, the deaths by cholera were only about equal to deaths of the same age by other causes.

In the week ending July 2d, the number of deaths by cholera, between 20 and 40, were 336, or much exceeding half of the whole number.

It is said that a very large portion of the deaths, the conjecture is about three-fourths, were foreigners, badly provided with the comforts of life, and perhaps many of them not having seasonable medical attendance.

